# Ecofriendly remediation of cadmium, lead, and zinc using dead cells of *Microcystis aeruginosa*

**DOI:** 10.1038/s41598-025-86884-1

**Published:** 2025-01-29

**Authors:** Mohamad S. Abdelkarim, Mohamed H. H. Ali, Doaa A. Kassem

**Affiliations:** 1 Hydrobiology Lab, National Institute of Oceanography and Fisheries (NIOF), Cairo, Egypt; 2 Chemistry Lab, National Institute of Oceanography and Fisheries (NIOF), Cairo, Egypt

**Keywords:** Ecology, Environmental sciences

## Abstract

The utilization of cyanobacteria toxin-producing blooms for metal ions adsorption has garnered significant attention over the last decade. This study investigates the efficacy of dead cells from *Microcystis aeruginosa* blooms, collected from agricultural drainage water reservoir, in removing of cadmium, lead, and zinc ions from aqueous solutions, and simultaneously addressing the mitigation of toxin-producing *M. aeruginosa* bloom. Some physical characterization of the dead biomass was performed, including scanning electron microscope (SEM) which revealed that, the cells form a dense, amorphous cluster, energy-dispersive X-ray (EDX) spectroscopy confirmed that carbon, oxygen, and nitrogen are the predominant elements in the biomass, Fourier transformation infrared (FTIR) spectroscopy identified several active function groups, including hydroxyl, aliphatic C–H amide I and amide II bands, carboxylate and carbonyl (C=O). Key factors influencing the adsorption process were examined. Under optimal conditions—pH 6, a biosorbent dose of 0.3 g, contact time of 90 min, primary metal level of 100 mg/L and temperature of 35 °C (313K)—a maximum removal efficiency exceeding 90% was achieved. Isothermal analysis revealed that the adsorption of Cd(II), Pb(II), and Zn(II) followed the Langmuir isotherm model (R^2^ = 0.96, *q*_max_ > 67 mg/g). Kinetic studies indicated that the pseudo-second-order model best described the adsorption process (R^2^ > 0.94 and *q*_*e*_ > 81.3 mg/g.), suggesting a dominant chemisorption mechanism. Thermodynamic analysis indicated that the adsorption process is spontaneous and endothermic. The findings highlight the potential of *M. aeruginosa* dead cells as a low-cost, sustainable biosorbent for the removal of heavy metal in wastewater treatment applications.

## Introduction

Human being and aquatic organisms are significantly impacted by cyanobacterial bloom^1^. *Microcystis* is responsible for some of the most extensive blooms worldwide^2^, recorded in over 108 countries in 2016^3^. Among them, *M. aeruginosa* is one of the most critical and extensively studied phytoplankton bloom. It can rapidly increase its biomass within short periods, producing massive biomass during bloom season. More than one million ton of wet weight of *Microcystis* spp have been collected annually in Lake Taihu^4^. These blooms pose serious problems in lakes and rivers, as their toxins and odorous compounds restrict water usage for drinking, irrigation, and aquaculture, while also posing severe risks to human health. *Microcystis aeruginosa* have unique characteristics with significant ecological and economic implications. This cyanobacterium is widely distributed, often forming blooms in freshwater ecosystems, and its abundance makes it an easily accessible natural biomass source. However, *M. aeruginosa* secret potent toxins called microcyctine, which have harmful impacts on both human and wildlife.

Nemours researchers have highlighted the detrimental impacts of *M. aeruginosa* blooms on the water quality, drinking water safety and the need for effective management strategies to mitigate these issues^5,6^. The cell walls of *M. aeruginosa* contain multi-functional groups such as hydroxyl, carboxyl, amide and amino groups, which serve as chelating agents for heavy metals thereby enhancing its biosorption capacity^7^. Furthermore, utilizing *M. aeruginosa* offers an eco-friendly and cost-effective solution for remediation of heavy metals. Its large-scale abundance in aquatic water body, resulting from dense blooms, provides a compelling rationale for utilizing its biomass as natural adsorbent, which lead to addressing the environmental challenges posed by such blooms. This approach not only supports sustainable and innovative environmental management but also transforms the harmful biological wastes of *M. aeruginosa* into a safe and sustainable resource. By repurposing this biomass into value-added products, it contributes to waste minimization and aligns with the principles of a circular economy.

The use of dead *Microcystis aeruginosa* biomass represents an innovative and sustainable eco-friendly technique for the remediation of toxic metals such as cadmium, lead and zinc. Unlike the conventional methods that are often expensive, consuming high energy and environmentally taxing, this study highlights the potential of biological wastes to remediate the heavy metals from polluted environment effectively. Furthermore, harnessing the natural biosorption properties of dead algal cells offers a green solution for mitigating heavy metal pollution, contributing to sustainable environmental management and advancing the field of bioremediation.

The cleanup of the contaminated environment with toxic heavy metals especially cadmium, and lead using innovative remediation strategies is a critical concern. The presence of these metals in aquatic ecosystem poses significant risks to both human health and the environment due to their highest toxicity. Cadmium is highly toxic element and associated with sever health issues such as kidney failure, lungs damage and osteoporosis^8^. Lead being non-biodegradable and highly toxic, impairs cognitive development, causes anaemia, liver dysfunction, neurological disorders and mental degradation^9^. Although zinc is an essential element necessary for various biological functions, but excessive levels can be harmful, leading to gastrointestinal distress and interfering with the adsorption of other essential minerals^10^.

Several strategies for heavy metals remediation has been developed, focusing on mitigating, neutralizing and eliminating pollution in various environments through a range of treatment techniques. Conventional approaches such as physical precipitation, chemical coagulation, and biological remediation have been widely utilized^11^. Despite, these methods have proven effective heavy metals remediation, they have significant disadvantages, including high costs, energy-intensive processes, production of toxic by-products and limited efficiency^12^. Recently, advanced remediation strategies have gained prominence, including bioremediation and phytoremediation using living organisms or their vital extracted components^13^. Nanoremediation, employing using green nanoparticles such as eco-friendly CuO NPs, is also, emerging as high effective method due to their large surface are which enhancing their reactivity^14^. These novel approaches offer numerous advantages, including increased efficiency, cost-effectiveness, reduced energy consumption, minimal toxic by-product generation and environmental sustainability. In recent decades, the integration and combination of these advanced strategies for remediation of heavy metal from various environments have become increasingly popular and essential^15^.

The use of dead algal cell represents a promising, eco-friendly and cost-effective approach for the radiation of heavy metals. Keryanti et al.^16^ reported that *Aphanothece* sp. removed 185.64 mg/g of Pb(II), while *Chaetoceros* sp. achieved removal capacity of 0.95 mg/g of Pb(II)^17^. Similarly, *Chlorella vulgaris* eliminated 23.3 mg/g of Pb(II)^18^, *Parachlorella* sp. removed 96.2 mg/g Cd(II)^19^ and *Scenedesmus*-24 removed 50.0 mg/g of Cd(II)^20^. Ramrakhiani et al.^21^ reported that dried activated tannery industry sludge biosorbent achieved a maximum removal efficiency exceeding 96% for Ni(II), Zn(II), Cd(II) and Co(II). Focusing on *Microcystis aeruginosa*, Cheraghpour et al.^22^ (2020) reported its ability to remove 20 mg/g of Cd(II) and 15 mg/g of Pb(II). Alwaleed et al^23^ (2021) demonstrated that removal of 80.5% (approximately 16mg/g) of Cd(II) from aqueous solution. Moreover, *M. aeruginosa* showed removal efficiency of 90–100% for Pb(II) and 79.5–100% for Cd(II)^24^. Chen et al.^25^ reported an 80% removal efficiency of Cd(II) and Pb(II).

Wadi El-Rayan is located approximately 125 km southwest of Cairo, covering an area of about 703 Km^2^. In the late 1960s, agricultural drainage from El Fayoum farmland was redirected to Wadi El-Rayan Depression after the capacity of Lake Qarun was exceeded. This drainage water eventually formed two lakes, known as the northern and the southern Lakes. The northern lake reached its maximum water level in 1978, covering approximately 5,100 hectares. Excess drainage water then flows through a shallow, swampy area into the southern lake^26^. Early in 1980s, a significant bloom of *M. aeruginosa* was recorded in the northern lake from late autumn to late winter^27,28^. The increased agricultural runoff has exacerbated the eutrophic conditions in this lake, creating an ideal environment for *M. aeruginosa* blooms.

Our study aims to (1) present an attempt to harness *M. aeruginosa* biomass collected form an agricultural drainage water reservoir for the sequestration of toxic heavy metals, including Cd(II), Pb(II), and Zn(II), (2) develop a cost-effective, eco-friendly, and sustainable method to reduce Cd(II), Pb(II), and Zn(II) levels in polluted water, (3) repurpose *M. aeruginosa* biomass to minimized environmental waste and mitigate the risks associated with its uncontrolled presence in water bodies, (4) achieve significant detoxification of environments polluted by heavy metal.

## Materials and methods

### Harvesting of algal biomass

**Fig. 1:**
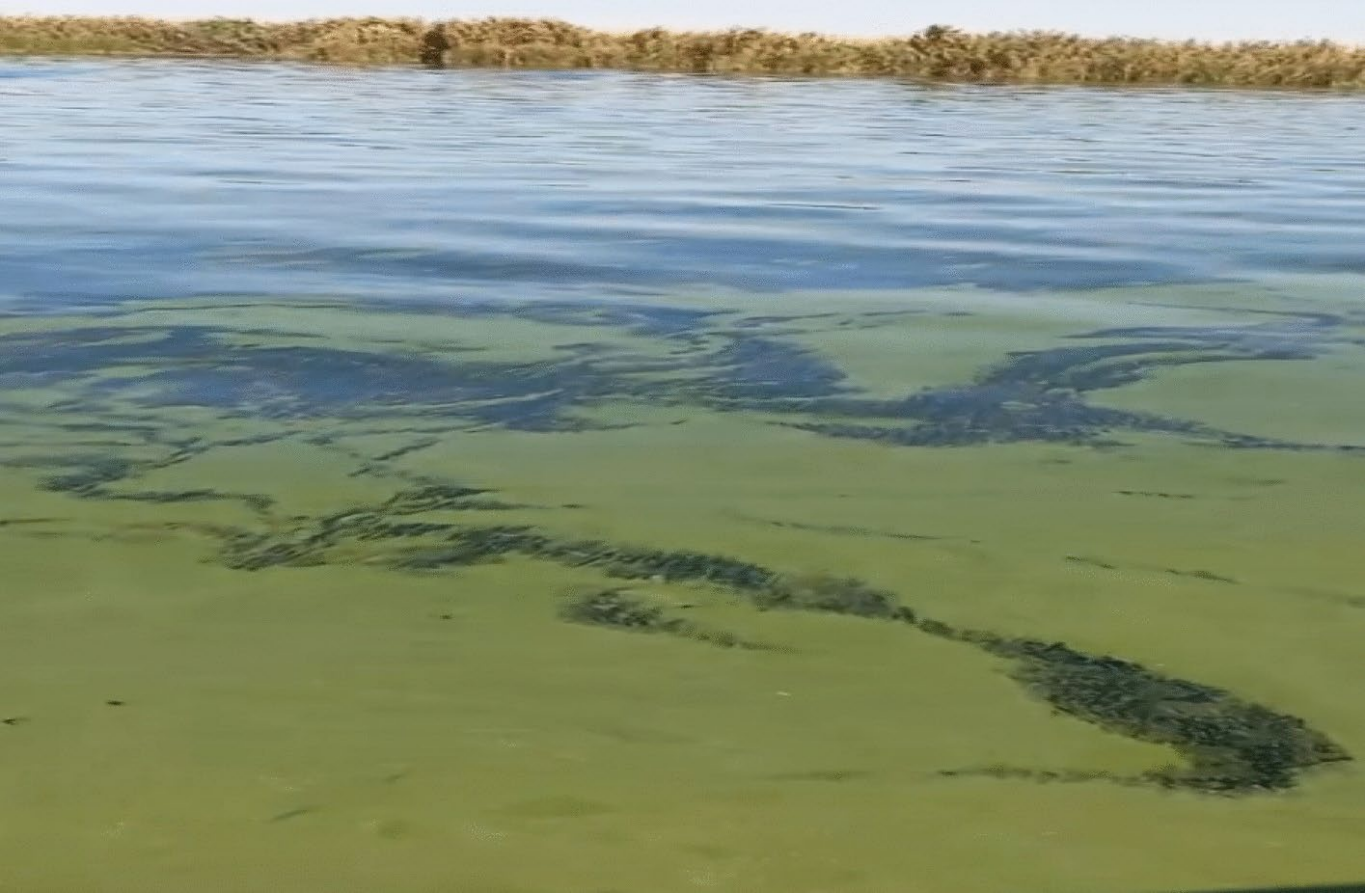
Bloom of *M. aeruginosa* in Wadi El-Rayan Lake during January 2024 (original photograph by the authors).

*Microcystis aeruginosa* cells were collected from Wadi El-Rayan Lakes, located in Western Desert, Egypt (29°11′50″N 30°24′30″E), during the winter bloom. Dense scums of *M*.* aeruginosa* were sampled in mid-January using 100µm mesh plankton net, filtrated through a 200 µm to remove large zooplankton and debris, and then concentrated using 20 µm in the field (Fig. 1, own photography). A natural sample unconcentrated sample was examined under inverted microscope, confirming that the scums primarily composed of *M. aeruginosa* colonies. The concentrated biomass was transported in ice boxes to the laboratory, where the algal cells were rinsed several times with distilled water, centrifuged, and dried in an air-forced oven (Memmert UE400) overnight at 60 °C. Once dried, the algal biomass was ground and sieved through a 63-mesh sieve, and stored in clean dry polyethylene bags for further uses.

### Chemicals and reagents

High-purity cadmium, lead and zinc salts were dissolved in deionized distilled water to prepare stock solutions, each with a concentration of 1000 mg/L. Cadmium nitrate (Cd(NO₃)₂), lead nitrate (Pb(NO₃)₂), and zinc nitrate Zn(NO₃)₂) were precisely weighed and dissolved. Serial standard solutions of the desired concentrations were then carefully prepared by diluting of the stock solutions. To ensure the reliability and accuracy of the results, clean glassware was used throughout the preparation process to prevent contamination.

### Physical characterization

The physical characterization of dead *Microcystis aeruginosa* cells included scanning electron microscopy (SEM), energy-dispersive X-ray spectroscopy (EDX) and Fourier transform infrared spectroscopy (FTIR), conducted both before and after the adsorption process. SEM and EDX analyses were performed using high-resolution scanning electron microscope, the Quanta field emission gun (FEG) equipped with an Oxford EDX system (model, FEL Quanta FEG 250 Instrument), to examine the surface morphology of the dead *M. aeruginosa* cells and observe any changes after the adsorption process. Additionally, EDX provided information about the elemental composition of the cells. FTIR analysis was carried out using an ATR-FTIR spectrometer (model, THERMO NICLOT, 50) within 400–4000 cm^−1^ range to identify the specific functional groups present in the cell wall or intracellular components.

### Adsorption experiments

Adsorption experiments were conducted to assess the adsorption efficiency and capacity of Cd(II), Pb(II) and Zn(II), selected based on their widespread environment occurrence, toxicity and their relevance to water pollution. The adsorption of these metals onto the surface of *M. aeruginosa* were investigated considering several key factors affecting the adsorption process. Initially, a series of solution with varying pH levels (3–9) were tested to explore pH effects on adsorption using 0.3 g of biosorbent for each experiment. Subsequently, different doses of *M. aeruginosa* biomass (0.05–0.35 g) were added to metal ions solution to assess the effect of biosorbent dosage on metal uptake. The examine how metal ion concentration gradients influences adsorption and to establish adsorption isotherm, experiments were conducted with initial concentrations of Pb(II), Cd(II), and Zn(II) ranging from 5 to 100 mg/L. The mixtures were agitated using magnetic stirrer at 300 rpm for specific time intervals (15 to 120 min) to observe the impact of time on adsorption. Finally, the adsorption process was carried out in different temperature (20 to 40 °C) to examine the temperature effect and to develop the thermodynamic adsorption models. Each series of tests was carried out in triplicate to ensure accuracy of the results. The experiments were allowed to reach equilibrium, after which, the solutions were filtered using Whatman No 1 filter paper. The residual metal concentrations were then measured by inductively coupled plasma mass spectrometry (Agilent 7900 ICP-MS). The adsorption percentage (R%) and adsorption capacity (*q*_*e*_ mg/g) under different experimental conditions were calculated using Eqs. 1 and 2.

Each test was carried out in triplicate to ensure consistency and accuracy of the results.

1 $$R\%=\frac{({C}_{0}-{C}_{e})}{{C}_{0}} x 100$$

2 $${q}_{e}= \frac{{(C}_{0}-{C}_{e}) x V}{M}$$

where, R%; is the removal percentage, Co and Ce; are the initial and final metal concentration, *q*_*e*_; is the adsorption capacity, M; is the biosorbent mass (g) and V the volume (L)

### Adsorption isotherm, kinetic and thermodynamic models

Adsorption isotherms, kinetic models, and thermodynamic studies are essential tools for understanding the adsorption process of heavy metals (Pb(II), Cd(II), and Zn(II)) onto the surface of dead *Microcystis aeruginosa* cells. Adsorption isotherms including Langmuir model (Eqs. 3 and 4), which assumes a monolayer adsorption of metal ions onto the biosorbent surface. It characterized by maximum adsorption capacity (q_*max*_ mg/g) and a constant (b) related to adsorption energy. The Freundlich model (Eq. 5), which describes the heterogeneous adsorption with Freundlich constant (K_f_) and an exponent (n) which indicates the adsorption intensity. Dubinin–Radushkevich (D–R) model (Eqs. 6 and 7, Table 1) which distinguishes between chemical and physical adsorption mechanisms.

**Table 1:** Mathematical equations used to calculate adsorption isotherm, kinetics and thermodynamics models of the studied metal ions on the *M. aeruginosa* biomass.

Models	Formula	Model key constants
*Adsorption isotherm models*		
Langmuir	$\frac{1}{{q}_{e}}=\frac{1}{{q}_{max}}+\frac{1}{b{q}_{max}}\cdot \frac{1}{{c}_{e}}$ (Eq. 3)	q_max_ (mg/g) the maximum metal ion uptake; *q*: concentration of metal ion (mg/g) at equilibrium; C_e_: mg/L initial metal concentration; and b (L/mg) Langmuir constant
	$${R}_{L}= \frac{1}{1+b{C}_{e}}$$ (Eq. 4)	
Freundlich	${q}_{e}={K}_{f}{C}_{e}^{1/n}$ (Eq. 5)	K_f_; the capacity of adsorbent, "n"; the intensity of adsorption
Dubinin and Radushkevich	$\text{ln}{q}_{e}=ln{q}_{m}-\upbeta {\varepsilon }^{2}$ (Eq. 6)	β; constant of D-R isotherm (mol^2^ kJ^−2^), ɛ: The Polanyi potential (kJ/mol) and E is the mean free energy
	$$E= \frac{1}{\sqrt{2\beta }}$$ (Eq. 7)	
*Kinetic models*		
Pseudo-first order	$\text{log}({C}_{e}-{C}_{t})=log{C}_{e}- \frac{{k}_{1}}{0.203} t$ (Eq. 8)	C_t_ and C_e_: the metal ions concentrations (mg/g) at time "t" and at equilibrium, respectively; k_1_ (min−^1^) and k_2_ (mg/g.min); constants rate of pseudo-first-order and pseudo-second-order reactions, respectively
Pseudo-second order	$\frac{t}{{C}_{t}}= \frac{1}{{k}_{2}{C}_{e}^{2}}+ \frac{1}{{C}_{e}} t$ (Eq. 9)	
*Thermodynamic models*		
Vant Hoff’s equation	$\Delta G= -RTlin {K}_{c}$ (Eq. 10)	R: the universal constant for gas (8.314 J mol−^1^K−^1^); Kc: equilibrium constant; T: temperature (Kelvin); Q_e_: metal concentration on the biosorbent (mg/g); C_e_: concentration of metal ion in the solution in equilibrium (mg/L). ΔG, ΔH° and ΔS° are free energy, entropy, respectively, and Kc: function of 1/T (Kelvin),
	$${K}_{c}=\raisebox{1ex}{${Q}_{e}$}\!\left/ \!\raisebox{-1ex}{${C}_{e}$}\right.$$ (Eq. 11)	
	$$Ln {K}_{c}=\frac{(\Delta^\circ \text{S})}{\text{R}}-\frac{(\Delta \text{H}^\circ )}{RT}$$ (Eq. 12)	

Kinetic studies involving the pseudo-first-order model (Eq. 8) suggests that the adsorption rate is proportional to the difference between the equilibrium concentration and the amount adsorbed, while the pseudo-second-order model (Eq. 9) proposes that the adsorption rate is depends on the square of the number of unoccupied sites.

Thermodynamic models (Eqs. 10–12, Table 1) evaluate the feasibility and spontaneity of the adsorption process through the parameters such as Gibbs free energy (ΔG), enthalpy (ΔH) and entropy (ΔS). The adsorption process is spontaneous if ΔG have negative value, if ΔH have negative value indicating exothermic adsorption process. The change of entropy (ΔS) reflects the degree of disorder associated with the adsorption process.

## Results and discussion

### Characterization

**Fig. 2:**
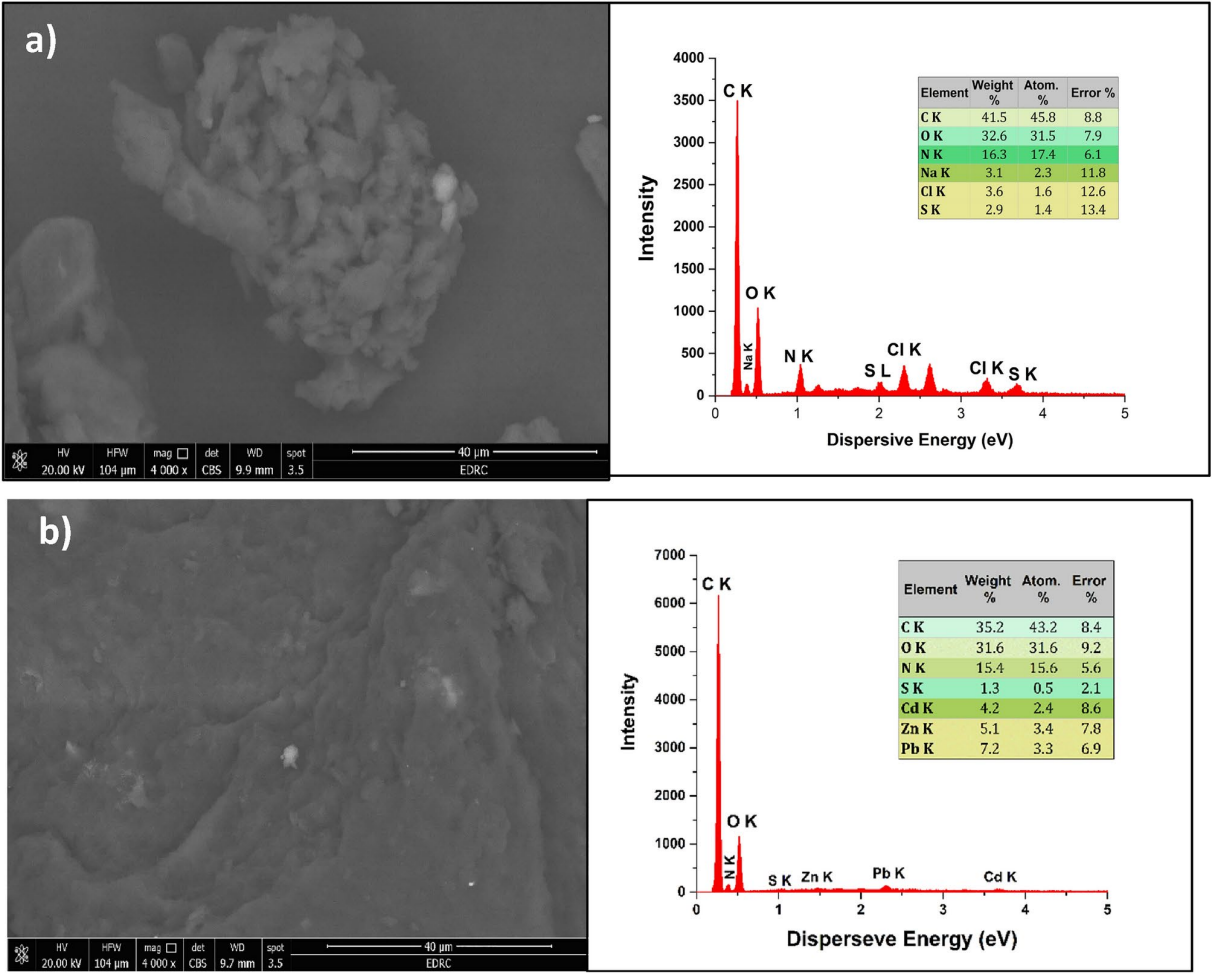
SEM and EDX images of dead *M. aeruginosa* cells (**a**) before and (**b**) after adsorption of metal ions.

The morphological features of the surface of dead *Microcystis aeruginosa* cells were characterized using SEM both before and after adsorption of metal ions (Fig. 2a,b). Prior to adsorption, SEM image reveals the cells are clustered together in a dense, amorphous mass typical of *M. aeruginosa* colonies. These cells are often embedded within a gelatinous matrix, which contributing to merged and blurry appearance of the surface structures^29^ (Fig. 2a). After metal adsorption, SEM images showed significant alterations in the surface of dead *M. aeruginosa* cells. The surface appeared uneven and rough, with visible aggregates and rough patches. Additionally, small, bright spots scattered across the surface are likely to be adsorbed metal precipitates, which appear brighter in the SEM image due to their higher atomic number compared to the biological material. Moreover, distinct regions where the metal particles are concentrated suggest that the adsorption process might not be uniform across the surface of the cells of *M. aeruginosa*^30,31^.

The EDX spectrum provides a detailed view of the elemental composition of *M. aeruginosa*. It confirms that carbon (41.5%), oxygen (32.6%), and nitrogen (16.3%) are the major elements in *M. aeruginosa* before metal adsorption (Fig. 2a), which consistent with the typical biochemical composition of cyanobacteria algae^23^. Additionally, minor peaks for sodium (3.1%), chloride (3.6%), and sulfur (2.9%) are also observed, which could be reflecting the sample’s environmental context. After adsorption of metal ions, three distinct small peaks according to Cd(II) (4.2%), Zn(II) (5.3) and Pb(II) (7.2%) appeared, confirming successful adsorption of these metal ions onto the surface of dead *M. aeruginosa* cells.

**Fig. 3:**
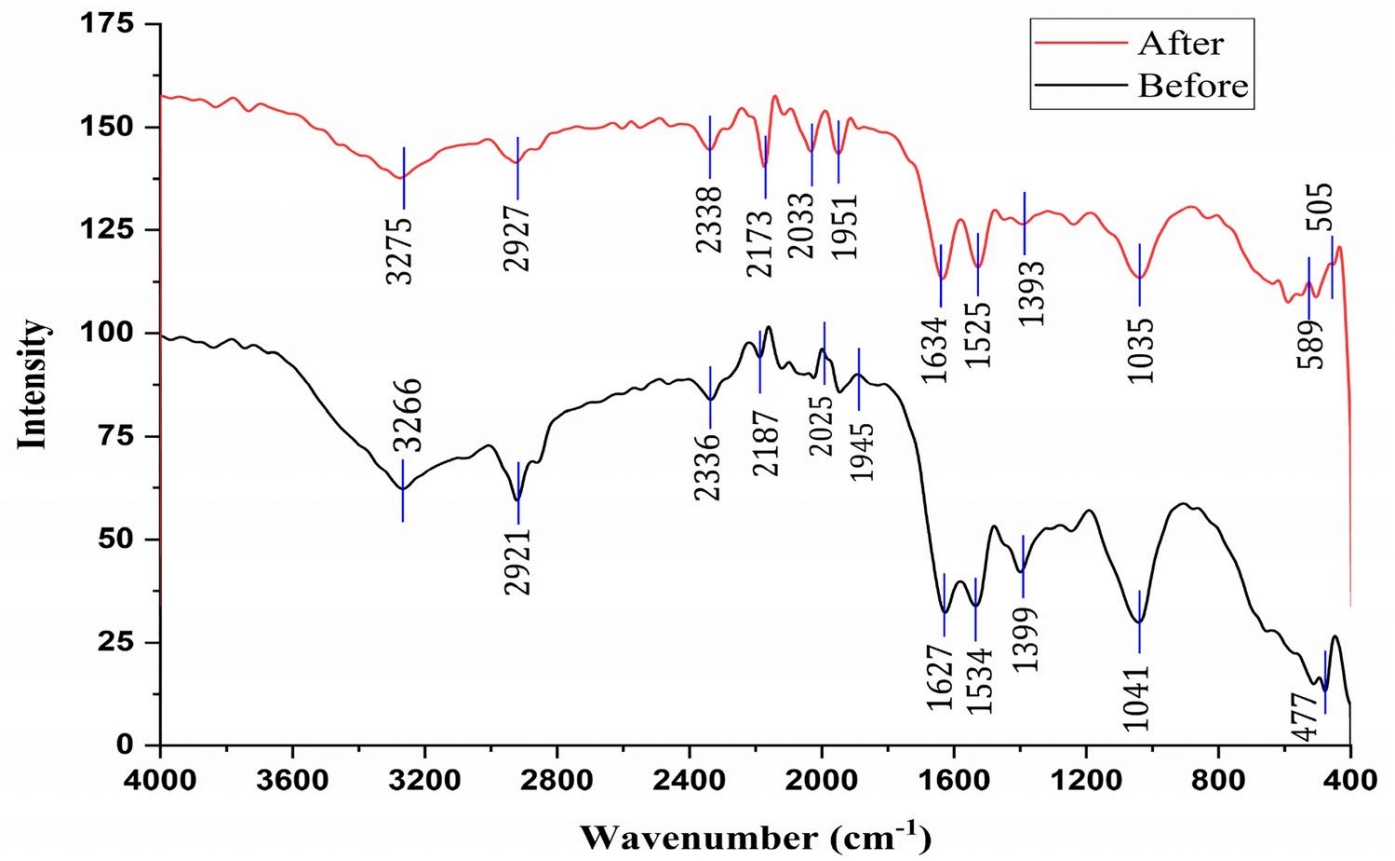
FTIR spectrum of dead *M. aeruginosa* cells before and after adsorption of metal ions.

Figure 3 and Table 2 present the FTIR spectrum of *M. aeruginosa* before and after the adsorption of Cd(II), Pb(II) and Zn(II), spanning from 4000 cm⁻^1^ to 400 cm⁻^1^. Before the adsorption, a prominent broad peak at 3266 cm⁻^1^ was observed, indicative to O–H and N–H stretching vibration, primarily associated with hydroxyl group in carbohydrate and amine groups in protein^32^. Another peak at 2921 cm⁻^1^ corresponds to aliphatic C–H stretching vibrations, which are related to lipid components within the cell membrane^33^. Additionally, strong absorption peaks at 1627 cm⁻^1^ and 1543 cm⁻^1^ are characteristic of amide I and amide II bands (N–H and C–N), representing the proteinaceous content of the cells^34^. Carboxylate groups, often associated with polysaccharides or proteins, exhibit strong peak at 1399 cm⁻^1^ due to carbonyl (C = O) stretching vibrations^32^. The peak at 1041 cm⁻^1^ is assigned to stretching vibrations of C–O and P–O (P = O) on polysaccharide and phospholipids, while the peak at 477 cm⁻^1^ is attributed to C≡C–H, C–H bend alkyne^35^.

**Table 2:** Shifts in the various functional group onto the *M. aeruginosa* surface before and after adsorption of Cd(II), Pb(II) and Zn(II).

Wavenumber (cm^−1^)		Attribution
Before adsorption	After adsorption	
3266.89	3275.79	O–H and N–H stretching vibration
2921.36	2927.36	aliphatic C–H stretching vibrations
1627.07	1634.8	amide I and amide II bands (N–H and C-N
1534.83	1525.6	
1399.51	1393.1	carbonyl (C = O) stretching vibrations
1041.4	1035.2	stretching vibrations of C-O and P-O (P = O)
477.71	505.26	C≡C–H, C–H bend alkyne

After adsorption, slight shift and change in their intensity were observed (Fig. 3, Table 2), the peak at 3266 cm⁻^1^ shifted slightly to 3275 cm⁻^1^, indicating changes in the O–H or N–H stretching vibrations due to interaction with metal ions. Also, the peak at 2921 cm⁻^1^ shifted to 2927 cm⁻^1^, possibly related to changes in the C–H stretching vibrations. Other shifts occur in the lower wavenumber region (below 1600 cm⁻^1^), potentially linked to metal ion binding to functional groups like carboxyl, amine, or hydroxyl groups. These spectral changes suggest that the adsorption of metal ions by *M. aeruginosa* leads to significant modifications in the chemical environment of the cell, particularly affecting functional groups involved in metal binding. The shifts and intensity changes in the spectra are indicative of the interaction between the biomass and the metal ions, resulting in the formation of new bonds or the alteration of existing ones^36^.

The mechanism of metals adsorption by *M. aeruginosa* largely despond on the presence of functional groups such hydroxyl (–OH), carboxyl (–COOH) and amide (–CONH) groups. These functional groups act as active binding sites which interact with metal ions through complexation, chelation and ions exchange process^37^. On the other hand, Ramrakhiani et al.^38^ found that the functional groups involved in adsorption of hexavalent chromium using inactivated fungal biomass of *Termitomyces clypeatus* followed this order of significance: carboxyl > phosphates > lipids > sulfhydryl > amines.

### Batch adsorption excitement

#### pH effect

**Fig. 4:**
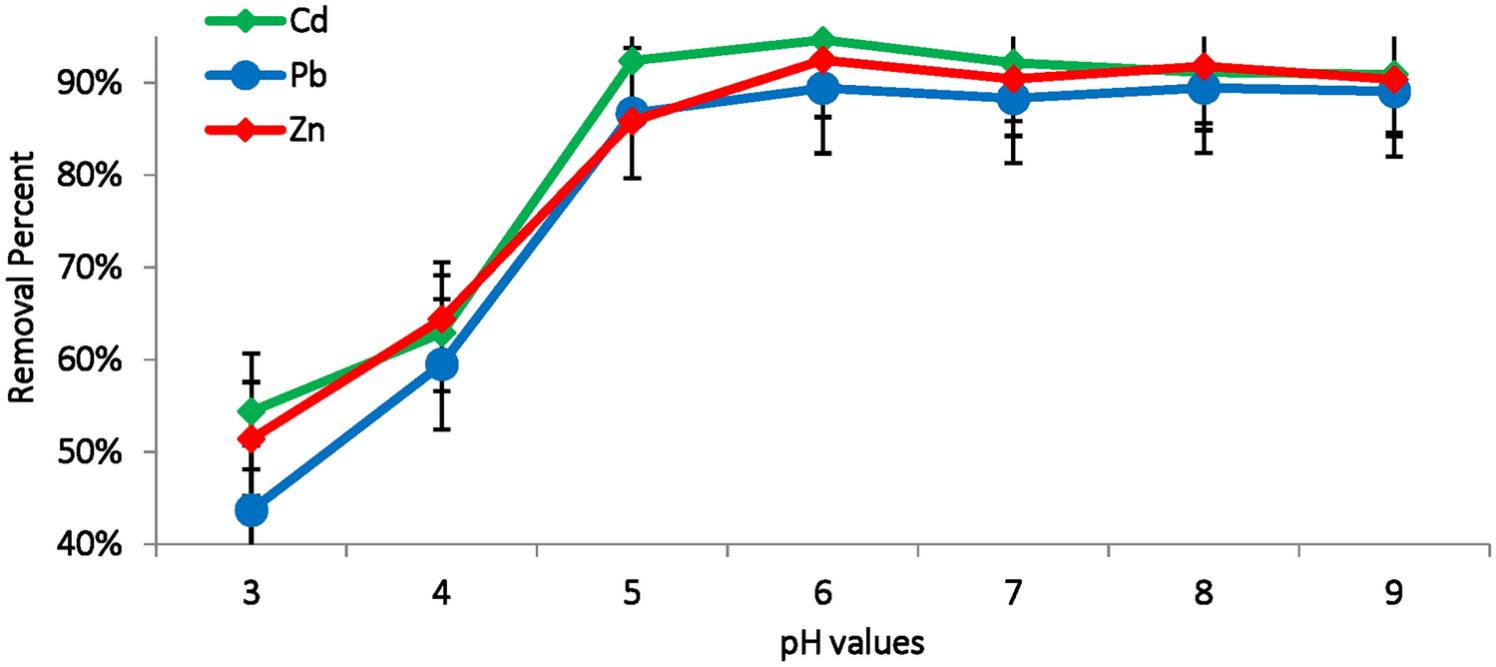
The impact of pH on the removal efficiency of Cd(II), Pb(II) and Zn(II) ions using *M. aeruginosa* biomass (biosorbent dose: 0.3g, temperature: 35°C, contact time: 90 min, metal concentration: 100 mg/L).

The pH of the solution is markedly influences adsorption of Cd(II), Pb(II) and Zn(II) onto the *M. aeruginosa* surface, since it affects the ionization state of both functional groups on the biosorbent surface, the speciation of metal ions in the solution and the electrostatic interaction between adsorbent and the metal ions^39^. At lower pH degrees (3–4), the surface of biosorbents tend to be more protonated, carrying more positive charges due to abundance of H^+^ ions leading to increasing the competition between positive metal ions and positively charged binding sites on the biosorbents surface, further lowering metal adsorption efficiency^40^. By increasing pH around natural condition (pH = 7), hydroxyl ions (OH^-^) increase, so the biomass surface become negatively charged, these negatively charged binding sites become more effective in binding with positively charged metal ions through electrostatic attraction and complexation. A low adsorption rate, ranging from 40 to 51% was observed at pH 3. However, the adsorption rate gradually increased with rising pH levels, reaching maximum of 89.4, 92.4 and 94.6% at pH 6 for Pb(II), Zn(II) and Cd(II) respectively (Fig. 4). Beyond pH 8, the adsorption efficiency decreased as metal ions began to precipitate as hydroxides rather than adsorb onto the biomass^41^. Keryanti et al.^16^ reported an optimum pH level of 7 for Pb(II) ions adsorption using *Aphanothece* sp., while Gu and Lan^42^ identified pH 6 as the optimum pH level for maximum adsorption of Cd(II) and Pb(II) ions using *Chlorella vulgaris* biomass. Similarly, Alwaleed et al.^23^ reported pH 6 as the optimal pH for maximum adsorption of Cd(II) ions using *M. aeruginosa* biomass.

#### Contact time effect

**Fig. 5:**
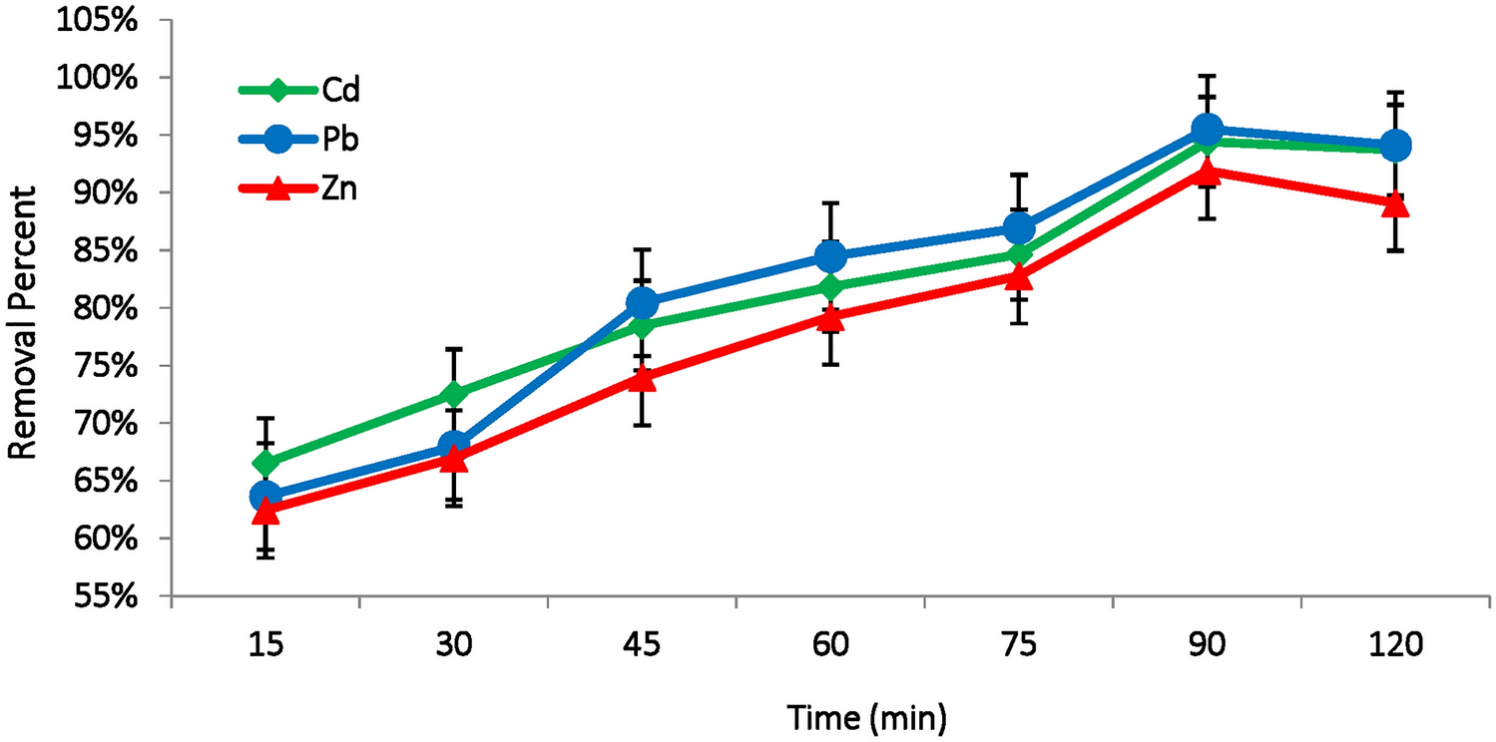
Effect of contact time (min) on the removal efficiency of Cd(II), Pb(II) and Zn(II) ions using *M. aeruginosa* biomass (pH:6, biosorbent dose: 0.3g, temperature: 35°C, metal concentration: 100 mg/L).

The effect pf contact time of on the removal efficiency of metal ions using different biosorbents, such as dead *M. aeruginosa* biomass is a crucial for optimizing the adsorption process^43^. At the start of the reaction, the adsorption rate of metal ions is rapid due to the abundance of available binding sites on the biomass surface. Metal ions quickly bind to accessible functional groups, such as hydroxyls, amides, amines, carboxyl, and phosphates. The results indicate that significant metal adsorption occurs within the first 15 min, reaching approximately 65% for the three studied metal ions. This increases to around 80% after 60 min, and reaches a maximum of 94.4%, 95.5% and 91.9% after 90 min for Cd(II), Pb(II) and Zn(II), respectively (Fig. 5).

Achieving maximum remediation within 90 min can be attributed to several stages, particularly the rapid initial uptake of metal ions by available binding sites, followed by slower kinetic process until equilibrium is reached. These processes are influenced by environmental conditions, such as pH, temperature and biomass characteristics. As contact time increases, the adsorption rate gradually slows down, reaching an equilibrium phase where the remaining metal ions in the solution compete for the limited number of partially occupied or unoccupied binding sites, leading to a reduced adsorption rate^44^.

Huang et al.^45^ reported that 30 min was the optimal contact time for highest removal of Cd(II) using dead biomass of *Bacillus cereus,* while Pardo et al.^46^ found that just 10 min were needed to remove 80% of Cu(II), Zn(II), Pb(II), and Cd(II) using dried biomass of *Pseudomonas putida*. (Table 3). Sometimes, higher contact time is required for maximum metal adsorption. Guo et al.^47^ reported that, the maximum Cd(II) ion adsorption using *Pseudomonas plecoglossicida* biomass occurred after 12 h.

**Table 3:** The removal efficiency, isothermal models and kinetic and of metal ions adsorption using various algal biomass adsorbents under specific condition.

Adsorbent	Adsorbate (metal ions)	Experiment conditions	Remediation Rate (%)	Isothermal models	Kinetic models	Refs
*Microcystis aeruginosa*	Cd(II), Pb(II)	pH = 7, 0.4 g dose	86–92	*	*	^22^
*Microcystis aeruginosa* (Living cell)	Al(III), Cd(II)	pH 7, time = 6 h	60–69%	*	*	^23^
*Microcystis aeruginosa* (Dead cell)	Al(III), Cd(II)	pH 7, time = 6 h	92.4–98.6%	*	*	^23^
*Microcystis aeruginosa*	Cd(II), Pb(II)	pH = 7, 3 h, 20 mg/L initial conc	90–100%	*	*	^24^
*Chlorella vulgaris*	Pb(II), Cd(II)	pH = 6, time = 10–30 min,	90%	Langmuir	Pseudo-second order	^42^
*Microcystis aeruginosa*	Sb(III)	pH = 4, time = 60 min, 0.4 g dose	90%	Freundlich & Langmuir	Pseudo-second order	^49^
*Microcystis biomass*	Sb(V)	pH = 2.5	85%	Freundlich	*	^54^
*Pseudomonas aeruginosa*	Cr(VI)	pH = 2, 120 min, 0.4 g dose, 100 mg/L concentrations	> 60%	Freundlich	pseudo second-order	^55^
Dead cells of *Microcystis aeruginosa*	Cd, Pb, Zn	100 mg/L, pH 6, 90 min	89 – 95%	Langmuir model	Pseudo-second order	Current Study

*: not available.

#### Biosorbent dosage effect

**Fig. 6:**
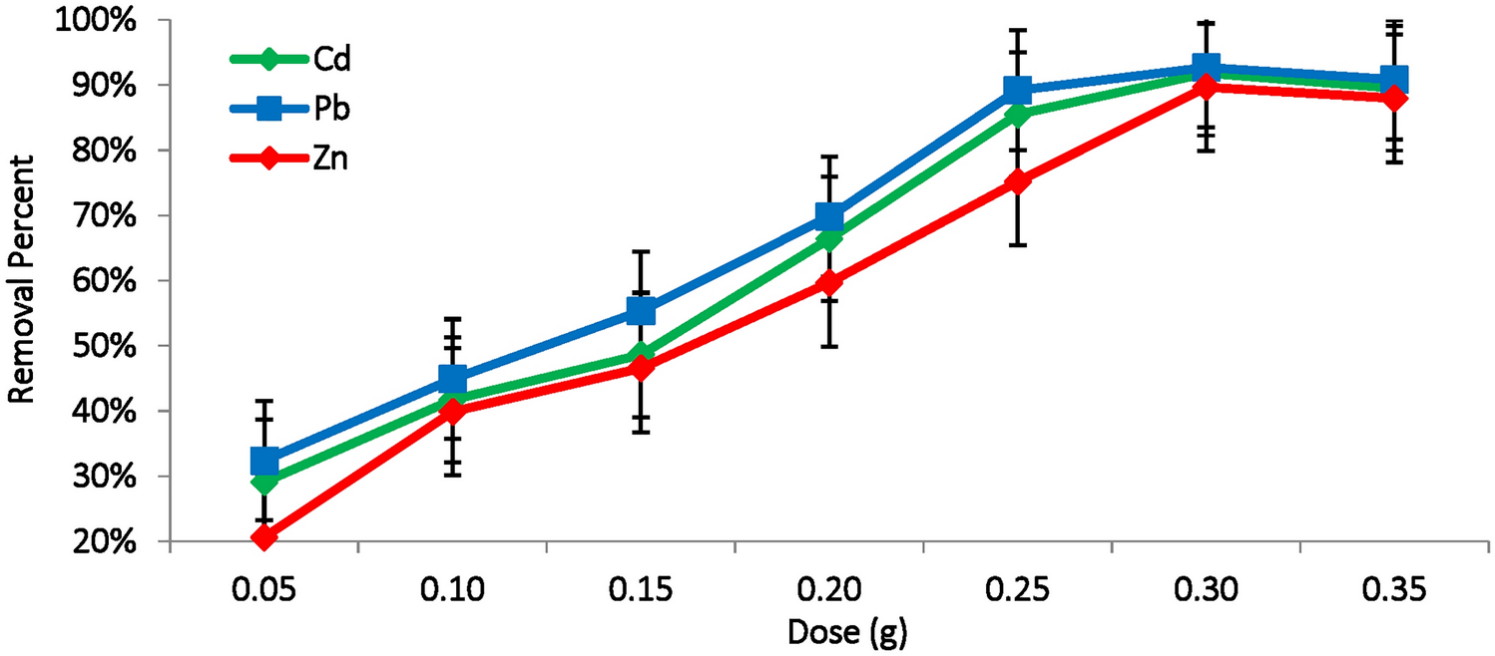
Effect of biosorbent dosage (g) on the removal efficiency of Cd(II), Pb(II) and Zn(II) ions using *M. aeruginosa* biomass (pH:6, contact time: 90min, temperature: 35°C, metal concentration: 100 mg/L).

The amount of *M. aeruginosa* biomass plays an essential role in the adsorption of metal ions, significantly influencing adsorption efficiency. Figure 6 illustrates the effect of *M. aeruginosa* dosage, ranging from 0.05 to 0.35 g, on the adsorption of Cd(II), Pb(II) and Zn(II). It is evident that as amount of dead *M. aeruginosa* biomass increases, the adsorption rate gradually rises. The adsorption of Zn(II), Cd(II), and Pb(II) increased from approximately 20%, 29% and 32% when using 0.05 g of biosorbent, reaching a maximum of 89.6%, 91.8% and 92.7%, respectively, at 0.3 g of biomass (Fig. 6). In general, increasing the biosorbent dosage provides a larger surface area and more available binding sites for metal ions, thereby enhancing metal uptake was occurred^48^. However, further increases in dosage often result in a notable decline in metal uptake, as higher dosage can lead to formation of aggregates, reducing the effective surface area available for adsorption. Wu et al.^49^ indicated that the maximum Sb(III) adsorption was achieved using 1.0 g of *Microcystis* biomass (Table, 3).

#### Effect of the initial metal concentration

**Fig. 7:**
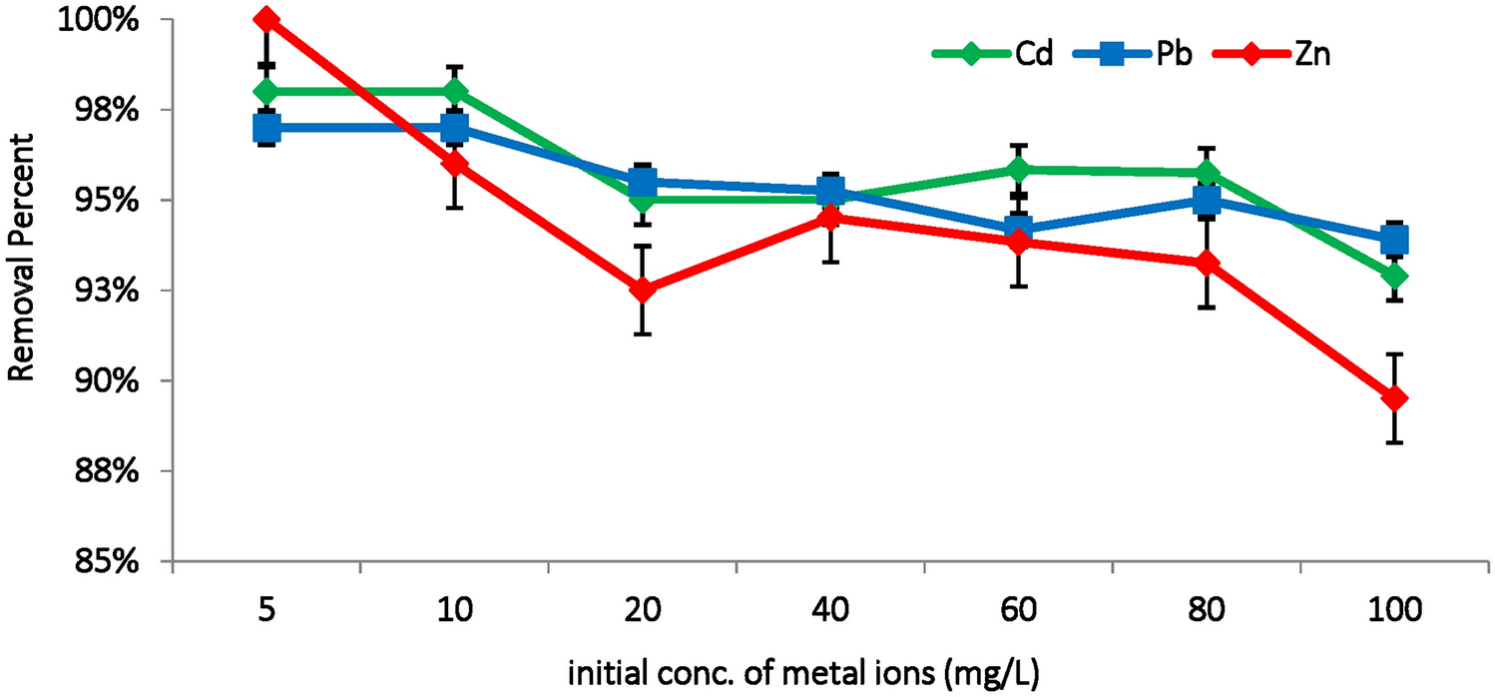
Effect of initial metal concentration (mg/L) on the removal efficiency of Cd(II), Pb(II) and Zn(II) ions using *M. aeruginosa* biomass (pH:6, biosorbent dose: 0.3g, temperature: 35°C, contact time: 90min).

The initial concentration of metal ions significantly influences the adsorption capacity of *M. aeruginosa* biomass, exhibiting a complex relationship characterized by both increased uptake and varying removal efficiencies^50^. As shown in Fig. 7 high adsorption efficiencies, nearly 100% were achieved at low metal concentrations (5, 10 mg/L). At these lower concentrations, the high ratio of available binding sites on the biomass surface relative to the number of metal ions enhances the adsorption efficiency (R%). This allows a large proportion of metal ions to be quickly captured by the various functional groups on the biomass, such as hydroxyl, amine, carboxyl, amide, and phosphate groups. However, as the initial metal concentration increases, competition among ions for the limited binding sites intensifies. This results in a higher absolute amount of metal ions being adsorbed, thereby increasing the adsorption capacity (*q*_*e*_), but the adsorption efficiency (R%) decrease as the biding sites become saturated. Notably, removal efficiencies (R%) decreased from 100% at lower concentrations to 89.5%, 92.9% and 93.9% for Zn(II), Cd(II), and Pb(II), respectively at 100mg/L (Fig. 7).

Optimizing the initial metal concentration is essential to balance maximizing metal removal while maintaining high efficiency. Gu and Lan^42^ identified 50mg/L as the optimal concentration for Pb(II) and Cd(II) adsorption onto the surface of dried *C. vulgaris* biomass. Similarly, Puranik, and Paknikar^50^ determined that approximately 25 mg/L was optimal for the adsorption of Cd(II), Pb(II), and Zn(II) using *Citrobacter* strain MCM B-181.

#### Effect of temperature

Temperature is an important factor influencing the adsorption of metal ions using algal biomass, as it impacts both the kinetic energy and the thermodynamic of the adsorption process^32^. An increase in temperature enhances the kinetic energy of the metal ions, allowing them to collide with and bind effectively to the available active sites on the biomass surface, potentially increasing the adsorption capacity^51^. Additionally, temperature changes affect the chemical interaction between metal ions and the existing function groups on the surface of biomass, strengthening the binding of metal ions to these functional groups, resulting in improved adsorption efficiency^52^.

**Fig. 8:**
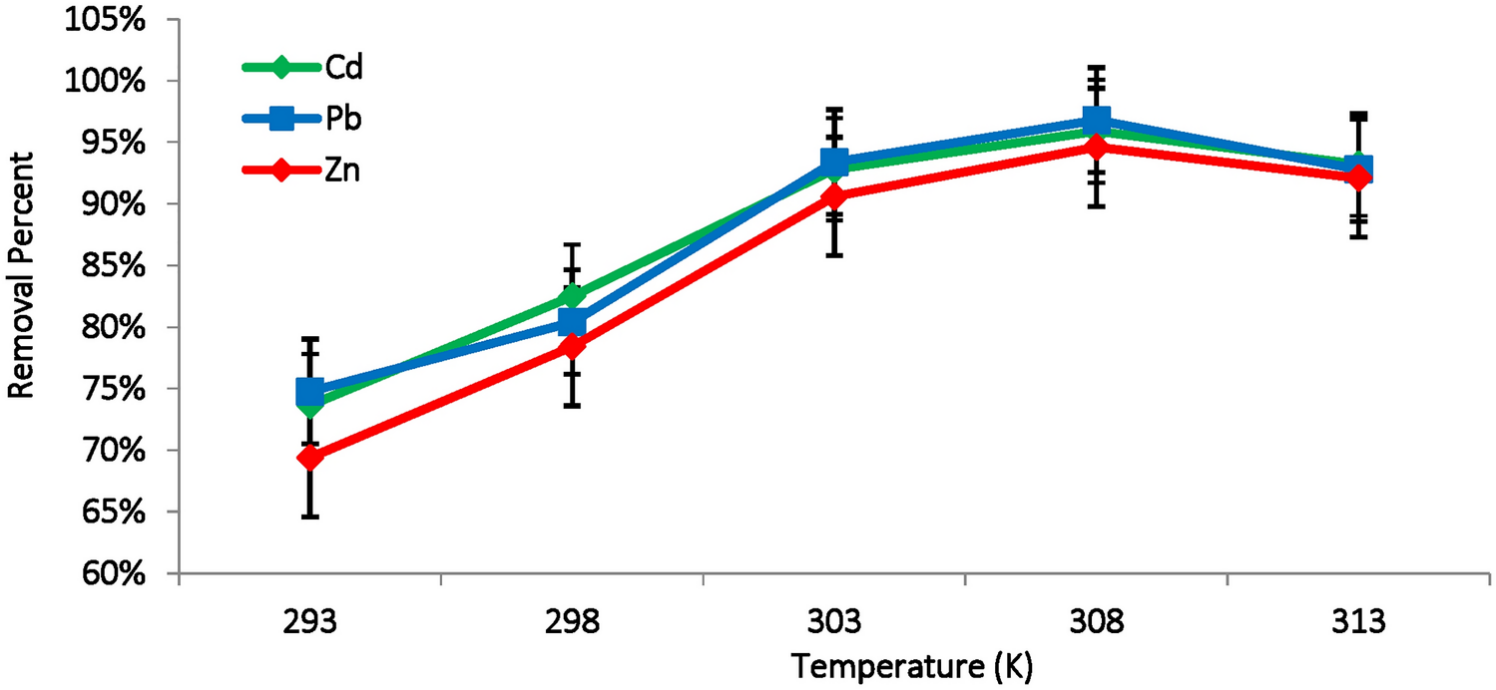
Effect of temperature (K) on the removal efficiency of Cd(II), Pb(II) and Zn(II) ions using *M. aeruginosa* biomass (pH:6, biosorbent dose: 0.3g, contact time: 90 min, metal concentration: 100 mg/L).

Figure 8 illustrates the effect of temperature, ranging from 20 to 40 °C (293–313 K) on the adsorption of Cd(II), Pb(II) and Zn(II) onto the surface of dead *M. aeruginosa* cells. A notable increase of the adsorption rate was observed with gradual rise in temperature, reaching maximum removal efficiencies of 94.6%, 95.9% and 96.8% for Zn(II), Cd(II) and Pb(II) respectively at 35 °C (308 K). Hlihor et al.^53^ reported that, the maximum adsorption of Cr(VI) (86%) was achieved at 40 °C using *Saccharomyces cerevisiae* biomass, while Zeng et al.^30^ identified a maximum absorption efficiency of 88.6% for Cd(II), Cu(II) and Ni(II) onto surface of *M. aeruginosa* was achieved at 35 °C.

### Isothermal models

Studying the isothermal models is essential for understanding the behavior of metal ions on the biosorbent biomasses. Three key models—Langmuir, Freundlich, and Dubinin–Radushkevich (D–R) isotherms—are commonly used to describe and optimize the adsorption process conditions.

**Fig. 9:**
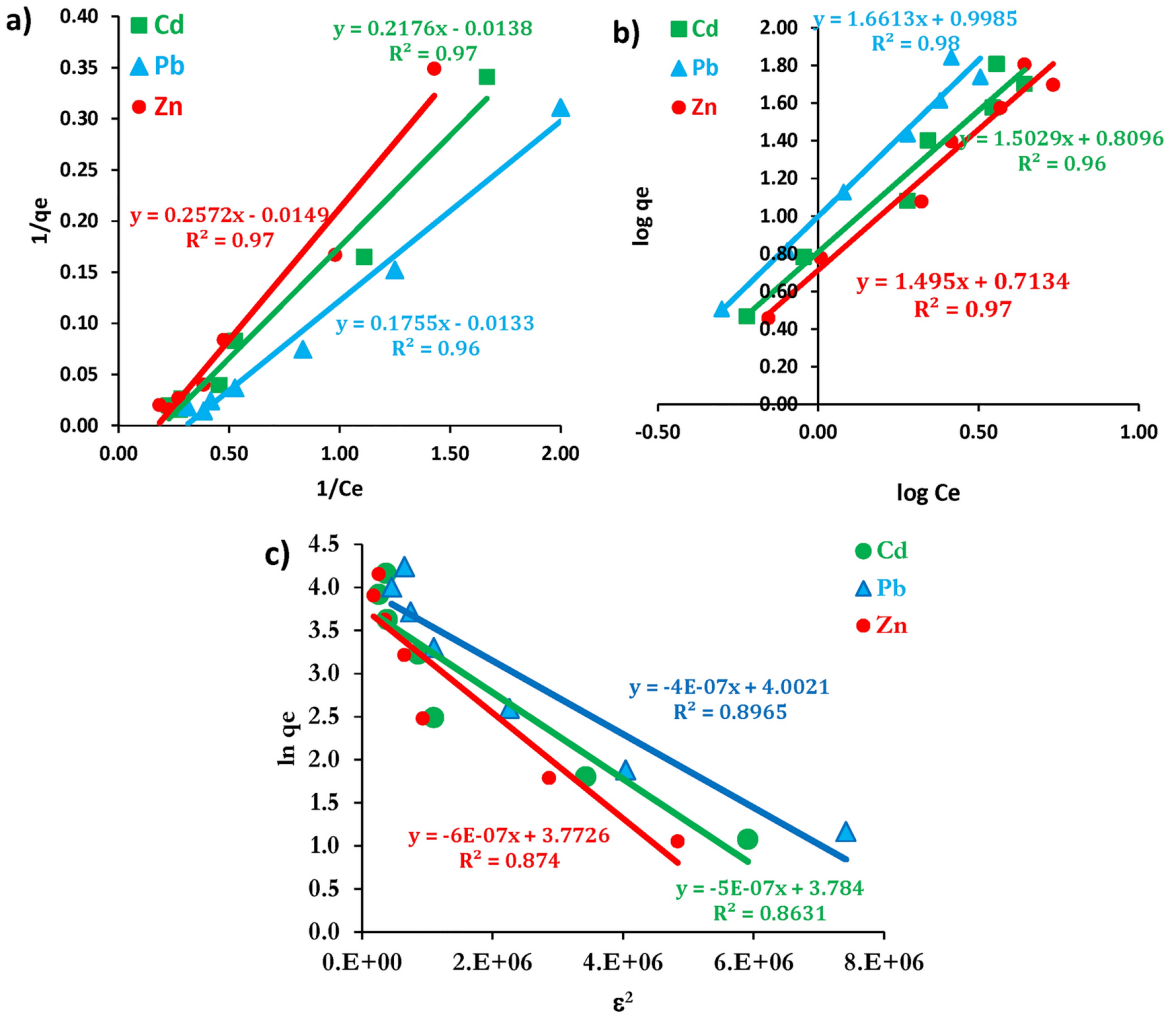
(**a**) Langmuir, (**b**) Freundlich and (**c**) Dubinin‒Radushkevich isotherms model for adsorption of Cd(II), Pb(II) and Zn(II) metal ions onto *M. aeruginosa* biosorbent.

The Langmuir isotherm model assumes monolayer adsorption, where each site on the biosorbent surface can accommodate only one adsorbate molecule (Eq. 3). The Langmuir constant (*b*, Eq. 4) defines the affinity between the biosorbent and adsorbate. Figure 9a shows the linear relationship between 1/*C*_*e*_ and 1/*q*_*e*_, with R^2^ greater than 0.96, indicating strong fit to the model. The maximum adsorption capacity (*q*_*max*_) were 67.11, 72.46 and 75.19 mg/g for Zn(II), Cd(II) and Pb(II) respectively. The separation factor (R_L_) of Langmuir model ranged from 0.03 to 0.05 (Table 4), falling within the range of 0 < R_L_ < 1, which indicates favorable adsorption process^56^.

**Table 4:** Calculated Key parameters constants of Langmuir, Freundlich and Dubinin Radushkevich isotherms.

Isotherm model	Parameter	Cd(II)	Pb(II)	Zn(II)
Langmuir	b (L/mg) *q*_*max*_ mg/g R_L_ R^2^	0.68	0.39	0.24
		46.95	47.02	47.17
		0.01	0.03	0.04
		0.989	0.992	0.990
Freundlich	K_f_ (mg/g) n R^2^	14.93	10.66	17.83
		1.60	1.55	1.56
		0.897	0.895	0.892
Dubinin Radushkevich	*q*_*max*_ mg/g E (kJ mol−1) R^2^	42.89	45.41	46.18
		74.5	84.5	79.1
		0.91	0.91	0.92

On the other hand, Freundlich model (Eq. 5) assumes heterogeneous adsorption with a non-uniform distribution of adsorption energy across the surface. Figure 9b illustrates the linear relationship between log *C*_*e*_ and log *q*_*e*_ with R^2^ value greater than 0.96. The Freundlich constant (*K*_*f*_) ranged from 5.17 to 9.9 mg/g, and the heterogeneity factor (*n*), was less than 1 (0.6–0.67), indicating less favorable adsorption as concentration increases, reflecting a non-uniform distribution of active sites on the biosorbent surface. Thus, the Langmuir model provided a better fit for the adsorption process compared with Freundlich model. Du et al.^57^ reported that, Langmuir isotherm model was the best-fit model for adsorption of Pb(II) and Cd(II) using *Pseudomonas putida* X4, with R^2^ values of ≥ 0.98. Similarly, Chakraborty et al.^58^ found that adsorption of Cd(II) using EPS extracted form *Pseudomonas aeruginosa* N6P6 also fit well with the Langmuir isotherm. Furthermore, the adsorption of Hg (II) onto surface of EPS from *Bacillus thuringiensis* PW-05 showed a good fit with the Langmuir model, with R^2^ values of ≥ 0.96^59^.

The Dubinin-Radushkevich (D-R) isotherm model (Eq. 6) focuses on the energy aspect of the adsorption process and is often applied to distinguish between physical and chemical adsorption^60^. The D-R model is represented in Fig. 9c, with its constant parameters listed in Table 4. The calculated mean free energy (E, Eq. 7) ranged between 913 – 1118 kJ mol^−1^ indicating that adsorption is likely chemical in nature, as E > 8 kJ/mol^61^.

### Kinetic models

**Fig. 10:**
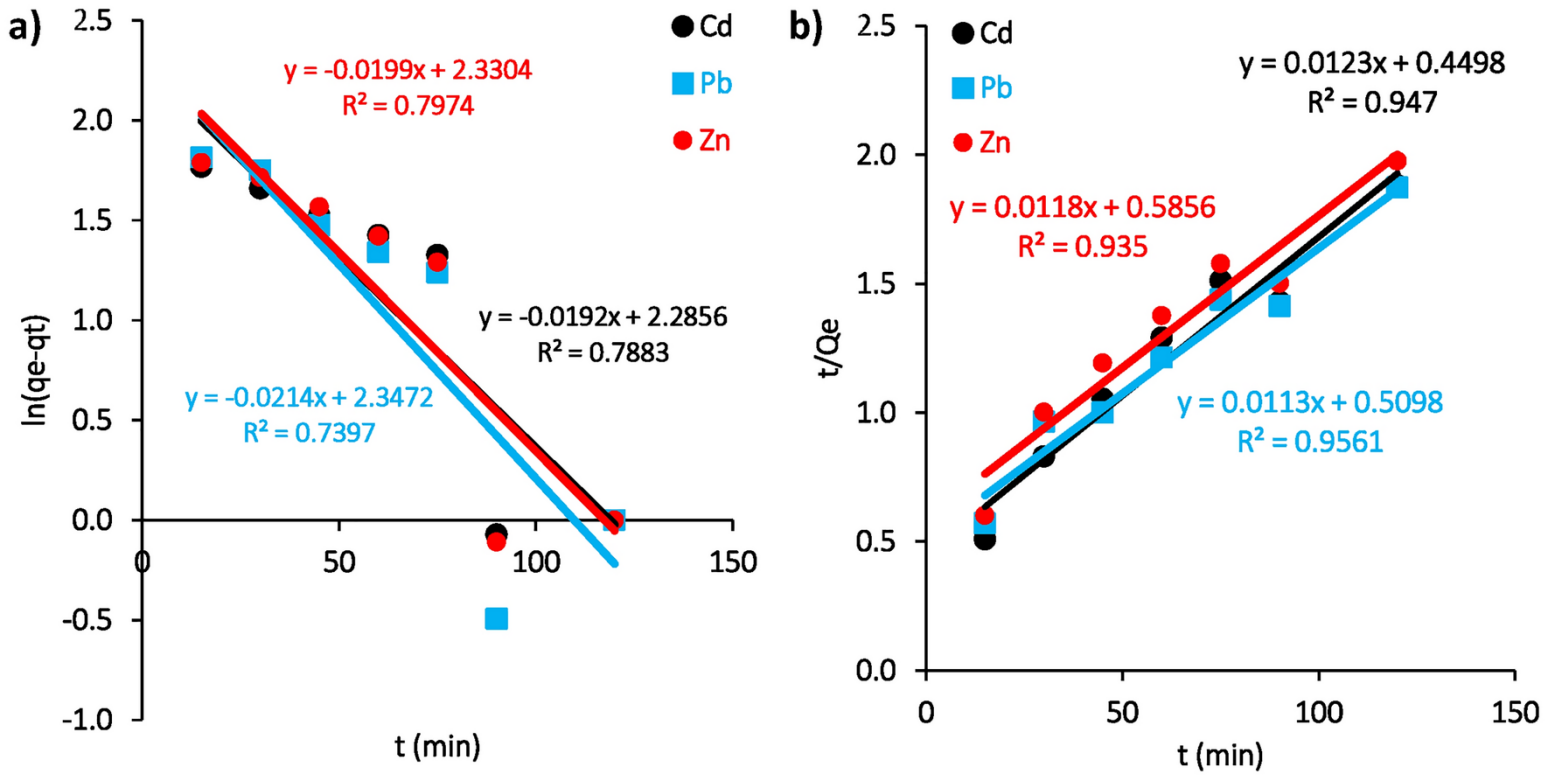
(**a**) Pseudo-first order kinetic and (**b**) Pseudo-second order kinetic models for adsorption of Cd(II), Pb(II) and Zn(II) onto *M. aeruginosa* biosorbent.

Studying the kinetic models of Cd(II), Pb(II), and Zn(II) adsorption onto the surface of *M. aeruginosa* biomass helps to elucidate the mechanisms and rate-controlling steps of the adsorption process. Two commonly used models, the pseudo-first-order (PFO, Eq. 8) and pseudo-second-order (PSO, Eq. 9) models, were applied. Figures 10 a,b depict the linear relationship for Cd(II), Pb(II) and Zn(II) adsorption onto the surface of dead *M. aeruginosa* biomass according to PFO and PSO kinetic models, while Table 5 presents the corresponding key constant parameters. The results indicate that, PSO model provides a better fit for the adsorption kinetic, with R^2^ ranging from 0.94 to 0.96 and *q*_*e*_ values between 81.3 to 88.49 mg/g. In contrast, PFO model showed R^2^ values ranged between 0.73 to 0.79 with *q*_*e*_ values ranging from 9.8 to 10.38 mg/g. Numerous studies have shown that the PSO model is more suitable for describing the adsorption of metal ions in various processes, such as Pb(II) using* Bacillus thioparans*^62^, Cr using *Bacillus salmalaya*^63^, Cd(II) using *Streptomyces* sp. K33 and HL-12^64^, Cu using Polyethyleneimine-bacterial cellulose bioadsorbent^65^ and Zn(II) using live and dead cells of *Streptomyces ciscaucasicus* strain CCNWHX 72–14^66^**.**

**Table 5:** Key kinetic parameters for PFO and PSO models for adsorption of Cd(II), Pb(II) and Zn(II) on *M. aeruginosa.*

Metal ion	Pseudo-first order reaction			Pseudo-second order reaction		
	*q*_*e*_ (mg/g)	K_1_ (min−^1^)	R^2^	*q*_*e*_ (mg/g)	K_2_ (g/mg min)	R^2^
Cd(II)	9.87	3.2E−04	0.78	81.30	3.4E−04	0.95
Pb(II)	10.38	1.8E−04	0.73	88.49	2.5E−04	0.96
Zn(II)	10.27	3.3E−04	0.79	84.74	2.4E−04	0.94

### Adsorption thermodynamics

**Fig. 11:**
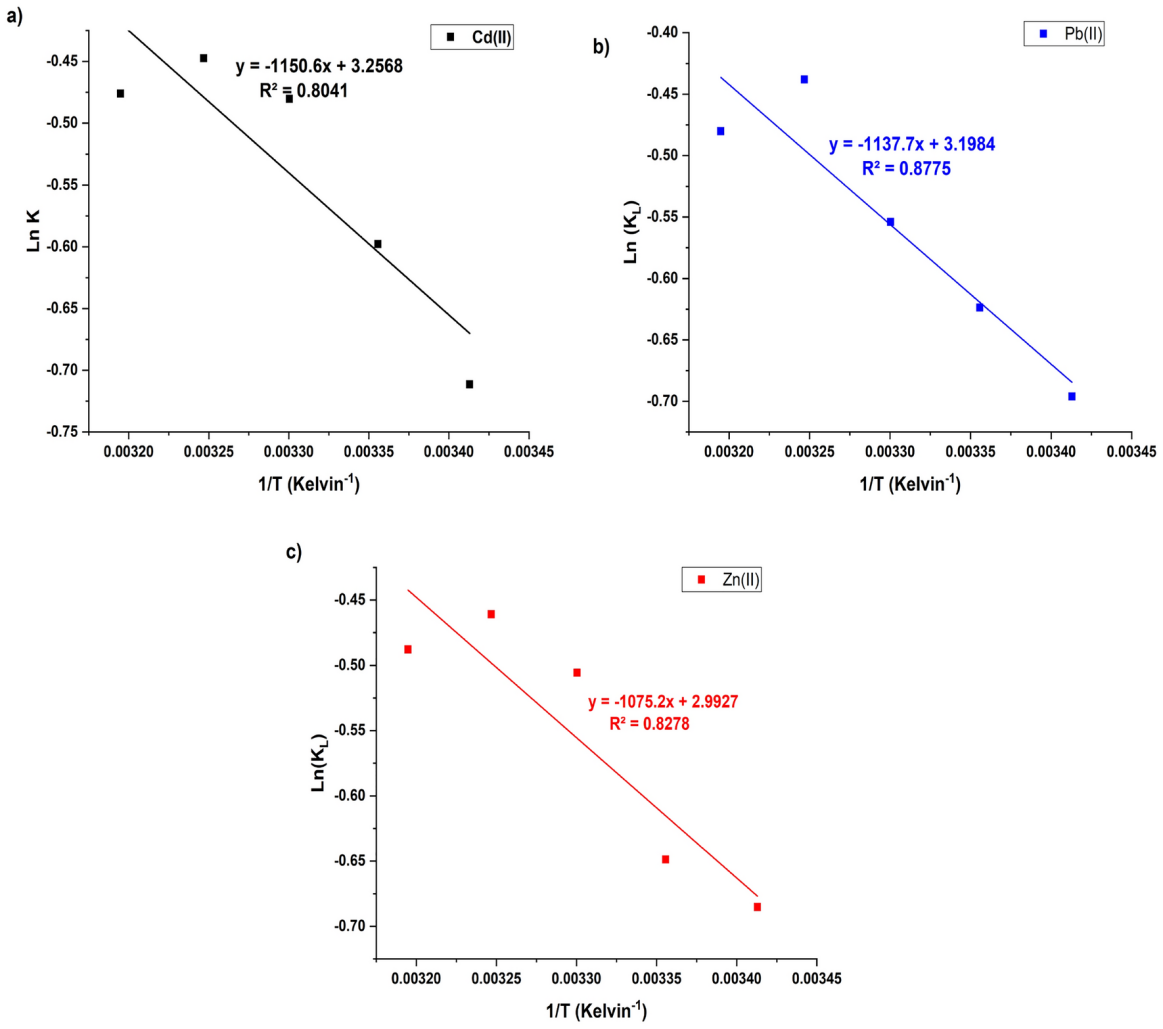
Thermodynamic plot for adsorption of; a) Cd(II), b) Pb(II) and c) Ni onto *M. aeruginosa* biomass**.**

The evolution of key thermodynamic parameters, including ΔG° (Gibbs free energy change), ΔH° (enthalpy change), and ΔS° (entropy change), helps to explore the heat changes, spontaneity, and disorder of the adsorption process. Figures 11 a–c depict the linear relation between ln(K_L_) and 1/T, with the key constants (∆H° and ΔS) calculated from the plot and listed in Table 6. The ΔG° values (from Eq. 10) are negative, ranging from − 1.73 to − 1.12 KJmol^−1^ for Cd(II), Pb(II) and Zn(II) (Table 6), indicating that the adsorption process is spontaneous and thermodynamically favorable at all temperatures. This suggests that the biosorbents biomass has high affinity for heavy metals ions greater than the surrounding solution, confirming that biosorbents efficiently captures and binds metal ions from the solution.

**Table 6:** Thermodynamic constants for the adsorption of Cd(II), Pb(II) and Zn(II) onto *M. aeruginosa* biomass at different temperatures (K).

	T (K)	K_L_	ΔH° (KJ mol−^1^)	ΔS° (JK−^1^ mol−^1^)	ΔG° (KJmol−^1^)	R^2^
Cd(II)	293	0.49	9.56	27.07	−1.73	0.80
	298	0.55			−1.48	
	303	0.62			−1.21	
	308	0.64			−1.15	
Pb(II)	313	0.62	9.45	26.59	−1.24	0.88
	293	0.50			−1.70	
	298	0.54			−1.55	
	303	0.57			−1.40	
Zn(II)	308	0.65	8.9	24.88	−1.12	0.82
	313	0.62			−1.25	
	293	0.50			−1.67	
	298	0.52			−1.61	

The enthalpy changes (∆H°) values are positive, ranging from 8.90 to 9.56 kJ mol^−1^ indicating that the process is endothermic. This means the adsorption process requires heat input and favored at higher temperatures^67^. Finally, the entropy change (ΔS°) ranging from 24.88 to 27.07 JK^−1^ mol^−1^ for the adsorption of Cd(II), Pb(II), and Zn(II) onto *M. aeruginosa* shows a low positive values indicating that the process is associated with an increase in randomness and the solid/solution interface, which reflects the strong ability of metal ions to be adsorbed onto the surface of the* M. aeruginosa* biosorbent. Overall, the thermodynamic analysis of the adsorption of Cd(II), Pb(II), and Zn(II) onto *M. aeruginosa* biomass suggests that the process is spontaneous, endothermic, and leads to an increase in disorder, making *M. aeruginosa* an effective biosorbent for these metals.

## Fate and reusability of the adsorbent biomass

The fate of the biosorbent mass used for the remediation of heavy metals is a critical aspect to consider for both environmental sustainability and potential health risks for humans. However, when these biosorbents are reused after adsorbing heavy metals, they may still contain residuals toxic metals that can pose threat to the environment through leaching if discarded without proper treatment. Therefore, these biosorbents must be regenerated or reused through various treatment methods to minimize environmental impacts. The choice of method depends on the type of the biomass and the desired outcome. Strict environmental regulations must be enforced to ensuring minimal risks. Several strategies can be employed, such as regenerating the biosorbents for further use in heavy metal removal or repurposing them for other applications, such as soil fertilizers. Alternatively, the biosorbents may be converted to biochar through controlled incineration process that prevent the release of harmful pollutants to atmosphere. Thus, a comprehensive assessment of the biosorbent’s fate and potential risks associated with its residues is essential for responsible waste management.

## Conclusion

The approach of harnessing the dead *Microcystis aeruginosa* cells for the remediation of cadmium (Cd II), lead (Pb II), and zinc (Zn(II) offers an effective, eco-friendly, sustainable and scalable solution, making it a promising option for large-scale environmental remediation. *M. aeruginosa* blooms are common in freshwater ecosystem and may pose environmental risks. Repurposing this biomass provides a cost effective way to manage algal overgrowth while addressing water pollution. Furthermore, using dead cells eliminates the need for costly cultivation and maintenance of live algae, further enhancing practicality. Physical characterization of biosorbent surface, including SEM imaging, reveals that the dead cells cluster together in a dense, amorphous mass. The EDX spectrum confirms that carbon, oxygen, and nitrogen are the predominant elements in *M. aeruginosa*. FTIR analysis identified several active function groups, such as O–H and N–H, hydroxyl, aliphatic C–H amide I and amide II bands, carboxylate and carbonyl (C = O). Spectral shifts observed after adsorption of metal ions. This study demonstrated that pH, biosorbent dosage, contact time, and temperature significantly affect the adsorption process. Under optimal conditions—pH 6, biosorbent dose of 0.3g, contact time of 90 min, initial metal concentration of 100 mg/L and temperature of 35 °C (313K)—the maximum removal efficiency exceeded 90%. Isothermal studies confirmed that Langmuir model best fits the adsorption process (R^2^ = 0.96, Q_max_ > 67 mg/g) compared to Freundlich model (R^2^ > 0.96, K_f_ ranging from 5.17 to 9.9 mg/g). Pseudo-second order reaction was more suitable for describing the adsorption of metal ions. Thermodynamic studies further supported the feasibility of this process, revealing that the adsorption of Cd(II), Pb(II), and Zn(II) onto *M. aeruginosa* is spontaneous and endothermic, with an increase in system entropy. The high bioremediation capacity of *M. aeruginosa* suggests promising prospects not only for establishing an environmentally sustainable strategy for cleaning up pollutants with a potential ‘polluter’, but also for utilizing the massive biomass of *M. aeruginosa* in more industrial applications.

## Data Availability

All data generated and analyzed during this study are included in the manuscript and are available from the corresponding author upon request.

## References

[1] Sha, J. et al. Harmful algal blooms and their eco-environmental indication. *Chemosphere* **274**, 129912. 10.1016/j.chemosphere.2021.129912 (2021). 33979937 10.1016/j.chemosphere.2021.129912

[2] Huang, J. et al. The magnitude and drivers of harmful algal blooms in China’s lakes and reservoirs: A national-scale characterization. *Water Res.* **181**, 115902. 10.1016/j.watres.2020.115902 (2020). 32505885 10.1016/j.watres.2020.115902

[3] Harke, M. J. et al. A review of the global ecology, genomics, and biogeography of the toxic cyanobacterium. *Microcystis spp. Harmful Algae.* **54**, 4–20. 10.1016/j.hal.2015.12.007 (2016). 28073480 10.1016/j.hal.2015.12.007

[4] Qin, B. et al. Why Lake Taihu continues to be plagued with cyanobacterial blooms through 10 years (2007–2017) efforts. *Sci. Bull.* **64**, 354–356. 10.1016/j.scib.2019.02.008 (2019). 10.1016/j.scib.2019.02.008 36659719

[5] Kong, Y. et al. Recent advances in the research on the anticyanobacterial effects and biodegradation mechanisms of *Microcystis aeruginosa* with microorganisms. *Microorganisms.* **10**(6), 1136. 10.3390/microorganisms10061136 (2022). 35744654 10.3390/microorganisms10061136 PMC9229865

[6] Qu, J. et al. Determination of the role of *Microcystis aeruginosa* in toxin generation based on phosphoproteomic profiles. *Toxins.* **10**(7), 304. 10.3390/toxins10070304 (2018). 30041444 10.3390/toxins10070304 PMC6070999

[7] Huang, I. S. & Zimba, P. V. Cyanobacterial bioactive metabolites—A review of their chemistry and biology. *Harmful Algae.* **86**, 139–209. 10.1016/j.hal.2018.11.008 (2019). 31358273 10.1016/j.hal.2019.05.001

[8] Kazantzis, G. Cadmium, osteoporosis and calcium metabolism. *Biometals.* **17**, 493–498. 10.1023/B:BIOM.0000045727.76054.f3 (2004). 15688852 10.1023/b:biom.0000045727.76054.f3

[9] Samuel, M. S., Shah, S. S., Bhattacharya, J., Subramaniam, K. & Singh, N. P. Adsorption of Pb (II) from aqueous solution using a magnetic chitosan/graphene oxide composite and its toxicity studies. *Int. J. Biol. Macromol.* **115**, 1142–1150. 10.1016/j.ijbiomac.2018.04.185 (2018). 29729343 10.1016/j.ijbiomac.2018.04.185

[10] Terrin, G. et al. Zinc in early life: A key element in the fetus and preterm neonate. *Nutrients.* **7**(12), 10427–10446. 10.3390/nu7125542 (2015). 26690476 10.3390/nu7125542 PMC4690094

[11] Samuel, M. S. et al. Nanomaterials as adsorbents for As(III) and As(V) removal from water: A review. *J. Hazard. Mater.* **424**, 127572. 10.1016/j.jhazmat.2021.127572 (2022). 34810009 10.1016/j.jhazmat.2021.127572

[12] Samuel, M. S. et al. Recent progress on the remediation of dyes in wastewater using cellulose-based adsorbents. *Indus. Crops Products.* **206**, 117590. 10.1016/j.indcrop.2023.117590 (2023).

[13] Ali, M. H., Abdelkarim, M. S., Mousa, I. E. & Belal, D. M. Extracted microbial exopolysaccharides: A sustainable approach to bioremediation of nickel, cobalt and copper ions; equilibrium, kinetics and thermodynamics studies. *Chem. Ecol.*10.1080/02757540.2024.2432877 (2024).

[14] Khedr, A. I. & Ali, M. H. Eco-friendly fabrication of copper oxide nanoparticles using peel extract of *Citrus aurantium* for the efficient degradation of methylene blue dye. *Sci Rep.* **14**, 29156. 10.1038/s41598-024-79589-4 (2024). 39587156 10.1038/s41598-024-79589-4 PMC11589848

[15] Deng, S., Zhang, X., Zhu, Y. & Zhuo, R. Recent advances in phyto-combined remediation of heavy metal pollution in soil. *Biotechnol. Adv.*10.1016/j.biotechadv.2024.108337 (2024). 38460740 10.1016/j.biotechadv.2024.108337

[16] Keryanti, K. & Mulyono, E. W. S. Determination of optimum condition of lead (Pb) biosorption using dried biomass microalgae Aphanothece sp. *Periodica Polytechnica*. *Chem. Eng.* **65**(1), 116–123. 10.3311/PPch.15773 (2021).

[17] Molazadeh, P., Khanjani, N., Rahimi, M. R. & Nasiri, A. Adsorption of Lead by Microalgae *Chaetoceros* Sp. and *Chlorella* Sp. from Aqueous Solution. *J. Commun. Heal. Res.* **4**, 114–127 (2015).

[18] Goher, M. E. et al. Biosorption of some toxic metals from aqueous solution using non-living algal cells of *Chlorella vulgaris*. *J. Elementol.* **21**(3), 703–714. 10.5601/jelem.2015.20.4.1037 (2016).

[19] Dirbaz, M. & Roosta, A. Adsorption, kinetic and thermodynamic studies for the biosorption of cadmium onto microalgae *Parachlorella* sp. *J. Environ. Chem. Eng.* **6**, 2302–2309. 10.1016/j.jece.2018.03.039 (2018).

[20] Jena, J. et al. Biological sequestration and retention of cadmium as CdS nanoparticles by the microalga *Scenedesmus*-24. *J. Appl. Phycol.* **27**, 2251–2260. 10.1007/s10811-014-0499-8 (2015).

[21] Ramrakhiani, L. et al. Industrial waste derived biosorbent for toxic metal remediation: Mechanism studies and spent biosorbent management. *Chem. Eng. J.* **308**, 1048–1064. 10.1016/j.cej.2016.09.145 (2017).

[22] Cheraghpour, J., Etemadifar, Z., Afsharzadeh, S. & Bahador, N. Assessment of bioremediation potential of *Microcystis aeruginosa* for removal of cadmium and lead ions from aqueous matrices. *Iran. J. Fish. Sci.* **19**(4), 1994–2009. 10.22092/IJFS.2018.119887 (2020).

[23] Alwaleed, E. A., Latef, A. A. A. & Mostafa, E. S. Biosorption efficacy of living and non-living algal cells of *Microcystis aeruginosa* to toxic metals. *Notulae Botanicae Horti Agrobotanici Cluj-Napoca.* **49**(1), 12149–12149. 10.15835/nbha49112149 (2021).

[24] Rzymski, P., Niedzielski, P., Karczewski, J. & Poniedziałek, B. Biosorption of toxic metals using freely suspended *Microcystis aeruginosa* biomass. *Open Chem.* **12**(12), 1232–1238. 10.2478/s11532-014-0576-5 (2014).

[25] Chen, J. Z., Tao, X. C., Xu, J., Zhang, T. & Liu, Z. L. Biosorption of lead, cadmium and mercury by immobilized *Microcystis aeruginosa* in a column. *Process Biochem.* **40**, 3675–3679. 10.1016/j.procbio.2005.03.066 (2005).

[26] Abdel Hameed, S., Ola, H., Imam, K. & Sherif, H. Correlation Between Algal Taxa and physico-chemical characters of the protected area of Wadi El-Rayan, Egypt. *Int. J. Agric. Biol.* **9**(1), 1–10 (2007).

[27] Konsowa, A. H. *Ecological studies on phytoplankton and productivity in the first Wadi El-Rayan Lake* (Thesis, Girls Collage, Ain Shams Univ, 1990).

[28] Ali, M. H., Abdel-Tawab, A. A., Ali, A. M. & Soliman, G. G. Monitoring of water quality and some pollutants of man-made lake (Wadi El-Rayan First Lake, Egypt). *Egyp. J. Aquat. Biol. Fish.* **11**(3), 1235–1251 (2007).

[29] Wang, B. et al. Efficient removal of U (VI) from aqueous solutions using the magnetic biochar derived from the biomass of a bloom-forming cyanobacterium (*Microcystis aeruginosa*). *Chemosphere* **254**, 126898. 10.1016/j.chemosphere.2020.126898 (2020). 32957293 10.1016/j.chemosphere.2020.126898

[30] Zeng, G. et al. Adsorption of heavy metal ions copper, cadmium and nickel by *Microcystis aeruginosa*. *Int. J. Environ. Res. Public Health.* **19**(21), 13867. 10.3390/ijerph192113867 (2022). 36360745 10.3390/ijerph192113867 PMC9656734

[31] Bayramoğlu, G., Tuzun, I., Celik, G., Yilmaz, M. & Arica, M. Y. Biosorption of mercury(II), cadmium(II) and lead(II) ions from aqueous system by microalgae *Chlamydomonas reinhardtii* immobilized in alginate beads. *Int. J. Miner. Process.* **81**(1), 35–43. 10.1016/j.minpro.2006.06.002 (2006).

[32] Saraeva, I. et al. FT-IR analysis of *P aeruginosa* bacteria inactivation by femtosecond IR laser radiation. *Int. J. Molecular Sci.* **24**(6), 5119. 10.3390/ijms24065119 (2023). 10.3390/ijms24065119 PMC10049678 36982184

[33] Yee, N., Benning, L. G., Phoenix, V. R. & Ferris, F. G. Characterization of metal-cyanobacteria sorption reactions: A combined macroscopic and infrared spectroscopic investigation. *Environ. Sci. Technol.* **38**, 775–782. 10.1021/es0346680 (2004). 14968864 10.1021/es0346680

[34] Quilès, F., Humbert, F. & Delille, A. Analysis of changes in attenuated total reflection FTIR fingerprints of *Pseudomonas fluorescens* from planktonic state to nascent biofilm state. *Spectrochimica Acta Part A Molecular Biomolecular Spectroscopy.* **75**(2), 610–616. 10.1016/j.saa.2009.11.026 (2010). 20004611 10.1016/j.saa.2009.11.026

[35] Yang, H., Wu, D., Li, H. & Hu, C. The extracellular polysaccharide determine the physico-chemical surface properties of Microcystis. *Front. Microbiol.* **14**, 1285229. 10.3389/fmicb.2023.1285229 (2023). 38125563 10.3389/fmicb.2023.1285229 PMC10732508

[36] Al-Qahtani, K. M., Abd Elkarim, M. S., Al-Fawzan, F. F., Al-Afify, A. D. & Ali, M. H. Biosorption of hexavalent chromium and molybdenum ions using extremophilic cyanobacterial mats: Efficiency, isothermal, and kinetic studies. *Int. J. Phytoremediation.* **26**(2), 228–240. 10.1080/15226514.2023.2232878 (2024). 37431240 10.1080/15226514.2023.2232878

[37] Al-Onazi, W. A., Ali, M. H. & Al-Garni, T. Using pomegranate peel and date pit activated carbon for the removal of cadmium and lead ions from aqueous solution. *J. Chem.* **2021**(1), 5514118. 10.1155/2021/5514118 (2021).

[38] Ramrakhiani, L., Majumder, R. & Khowala, S. Removal of hexavalent chromium by heat inactivated fungal biomass of *Termitomyces clypeatus*: Surface characterization and mechanism of biosorption. *Chem. Eng. J.* **171**(3), 1060–1068. 10.1016/j.cej.2011.05.002 (2011).

[39] Ali, M. H., Abd Elkarim, M. S., Haroun, S. & Attwa, K. Bioremediation of Fe, Zn(II) and Cd(II) ions from aqueous solution using died cells of cyanobacterial mats from extreme habitat, Siwa Oasis, Egypt. *Egypt. J. Aquat. Biol. Fish.* **22**(5), 511–522. 10.21608/EJABF.2019.25914 (2019).

[40] Al-Qahtani, K. M., Ali, M. H., Abdelkarim, M. S. & Al-Afifi, A. D. Efficiency of extremophilic microbial mats for removing Pb(II), Cu(II), and Ni(II) ions from aqueous solutions. *Environ. Sci. Pollut. Res.* **28**, 53365–53378. 10.1007/s11356-021-14571-5 (2021). 10.1007/s11356-021-14571-5 34031835

[41] Ali, M. H. et al. The isotherm and kinetic studies of the biosorption of heavy metals by non-living cells of *Chlorella vulgaris*. *J. Elementol.* **21**(4), 1263–1276. 10.5601/jelem.2016.21.1.1040 (2016).

[42] Gu, S. & Lan, C. Q. Lipid-extraction algal biomass for biosorption of bivalent lead and cadmium ions: Kinetics and isotherm. *Chem. Eng. Sci.* **276**, 118778. 10.1016/j.ces.2023.118778 (2023).

[43] Hussein, S. H., Qurbani, K., Ahmed, S. K., Tawfeeq, W. & Hassan, M. Bioremediation of heavy metals in contaminated environments using *Comamonas* species: A narrative review. *Bioresource Technol. Rep.*10.1016/j.biteb.2023.101711 (2023).

[44] Danouche, M., ElGhachtouli, N. & El Arroussi, H. Phycoremediation mechanisms of heavy metals using living green microalgae: Physicochemical and molecular approaches for enhancing selectivity and removal capacity. *Heliyon*10.1016/j.heliyon.2021.e07609 (2021). 34355100 10.1016/j.heliyon.2021.e07609 PMC8322293

[45] Huang, F. et al. Biosorption of Cd (II) by live and dead cells of Bacillus cereus RC-1 isolated from cadmium-contaminated soil. *Colloids Surf B Biointerfaces* **107**, 11–18. 10.1016/j.colsurfb.2013.01.062 (2013). 23466537 10.1016/j.colsurfb.2013.01.062

[46] Pardo, R., Herguedas, M., Barrado, E. & Vega, M. Biosorption of cadmium, copper, lead and zinc by inactive biomass of *Pseudomonas putida*. *Anal. Bioanal. Chem.* **376**, 26–32. 10.1007/s00216-003-1843-z (2003). 12734614 10.1007/s00216-003-1843-z

[47] Guo, J., Zheng, X. D., Chen, Q. B., Zhang, L. & Xu, X. P. Biosorption of Cd (II) from aqueous solution by *Pseudomonas plecoglossicida*: Kinetics and mechanism. *Curr. Microbiol.* **65**, 350–355. 10.1007/s00284-012-0164-x (2012). 22706778 10.1007/s00284-012-0164-x

[48] Priyadarshanee, M. & Das, S. Biosorption and removal of toxic heavy metals by metal tolerating bacteria for bioremediation of metal contamination: A comprehensive review. *J. Environ. Chem. Eng.* **9**(1), 104686. 10.1016/j.jece.2020.104686 (2021).

[49] Wu, F., Sun, F., Wu, S., Yan, Y. & Xing, B. Removal of antimony (III) from aqueous solution by freshwater cyanobacteria Microcystis biomass. *Chem. Eng. J.* **183**, 172–179. 10.1016/j.cej.2011.12.050 (2012).

[50] Puranik, P. R. & Paknikar, K. M. Biosorption of lead, cadmium, and zinc by *Citrobacter* strain MCM B-181: Characterization Studies. *Biotechnol. Prog.* **15**(2), 228–237. 10.1021/bp990002r (1999). 10194398 10.1021/bp990002r

[51] Kanamarlapudi, S. L. R. K., Chintalpudi, V. K. & Muddada, S. Application of biosorption for removal of heavy metals from wastewater. *Biosorption.* **18**(69), 70–116. 10.5772/intechopen.77315 (2018).

[52] Raji, Z., Karim, A., Karam, A. & Khalloufi, S. Adsorption of heavy metals: Mechanisms, kinetics, and applications of various adsorbents in wastewater remediation—a review. *Waste.* **1**(3), 775–805. 10.3390/waste1030046 (2023).

[53] Hlihor, R. et al. Bioremediation of Cr (VI) polluted wastewaters by sorption on heat inactivated *Saccharomyces cerevisiae* biomass. *Int. J. Environ. Res.* **7**, 581–594 (2013).

[54] Sun, J., He, X., Yilin, L. E., Al-Tohamy, R. & Ali, S. S. Potential applications of extremophilic bacteria in the bioremediation of extreme environments contaminated with heavy metals. *J. Environ. Manage.* **352**, 120081. 10.1016/j.jenvman.2024.120081 (2024). 38237330 10.1016/j.jenvman.2024.120081

[55] Ojiagu, K. D., Odibo, F. J. C., Ojiagu, N. C., Agu, K. C. & Okafor, A. C. Biosorption of hexavalent chromium by *Pseudomonas aeruginosa* strain ANSC: Equilibria isothermic, kinetic and thermodynamic studies. *Bioeng. Biosci.* **6**(1), 1–10. 10.13189/bb.2018.060101 (2018).

[56] Dada, A. O., Olalekan, A. P., Olatunya, A. M. & Dada, O. Langmuir, Freundlich, Temkin and Dubinin–Radushkevich isotherms studies of equilibrium sorption of Zn^2+^ unto phosphoric acid modified rice husk. *IOSR J. Appl. Chem.* **3**(1), 38–45. 10.9790/5736-0313845 (2012).

[57] Du, H. et al. Competitive adsorption of Pb and Cd on bacteria–montmorillonite composite. *Environ. Pollut.* **218**, 168–175. 10.1016/j.envpol.2016.08.022 (2016). 27566847 10.1016/j.envpol.2016.08.022

[58] Chakraborty, J., Mallick, S., Raj, R. & Das, S. Functionalization of extracellular polymers of Pseudomonas aeruginosa N6P6 for synthesis of CdS nanoparticles and cadmium bioadsorption. *J. Polym. Environ.* **26**, 3097–3108. 10.1007/s10924-018-1195-6 (2018).

[59] Dash, H. R. & Das, S. Interaction between mercuric chloride and extracellular polymers of biofilm-forming mercury resistant marine bacterium *Bacillus thuringiensis* PW-05. *RSC Adv.* **6**(111), 109793–109802. 10.1039/C6RA21069D (2016).

[60] Hu, Q. & Zhang, Z. Application of Dubinin–Radushkevich isotherm model at the solid/solution interface: A theoretical analysis. *J. Molecular Liq.* **277**, 646–648. 10.1016/j.molliq.2019.01.005 (2019).

[61] Sarı, A., Tuzen, M., Cıtak, D. & Soylak, M. Adsorption characteristics of Cu (II) and Pb (II) onto expanded perlite from aqueous solution. *J. Hazard. Mater.* **148**(1–2), 387–394. 10.1016/j.jhazmat.2007.02.052 (2007). 17386972 10.1016/j.jhazmat.2007.02.052

[62] Rodríguez-Tirado, V., Green-Ruiz, C. & Gómez-Gil, B. Cu and Pb biosorption on *Bacillus thioparans* strain U3 in aqueous solution: Kinetic and equilibrium studies. *Chem. Eng. J.* **181**, 352–359. 10.1016/j.cej.2011.11.091 (2012).

[63] Dadrasnia, A., Chuan Wei, K. S., Shahsavari, N., Azirun, M. S. & Ismail, S. Biosorption potential of Bacillus salmalaya strain 139SI for removal of Cr (VI) from aqueous solution. *Int. J. Environ. Res. Public Health* **12**(12), 15321–15338. 10.3390/ijerph121214985 (2015). 26633454 10.3390/ijerph121214985 PMC4690921

[64] Yuan, H. P., Zhang, J. H., Lu, Z. M., Min, H. & Wu, C. Studies on biosorption equilibrium and kinetics of Cd2+ by *Streptomyces* sp K33 and HL-12. *J. Hazard. Mater.* **164**(2–3), 423–431. 10.1016/j.jhazmat.2008.08.014 (2009). 18809250 10.1016/j.jhazmat.2008.08.014

[65] Jin, X., Xiang, Z., Liu, Q., Chen, Y. & Lu, F. Polyethyleneimine-bacterial cellulose bioadsorbent for effective removal of copper and lead ions from aqueous solution. *Bioresource Technol.* **244**, 844–849. 10.1016/j.biortech.2017.08.072 (2017). 10.1016/j.biortech.2017.08.072 28841789

[66] Li, H. et al. Biosorption of Zn (II) by live and dead cells of *Streptomyces ciscaucasicus* strain CCNWHX 72–14. *J. Hazard. Mater.* **179**(1–3), 151–159. 10.1016/j.jhazmat.2010.02.072 (2010). 20307931 10.1016/j.jhazmat.2010.02.072

[67] Zhang, W. et al. Enhanced heavy metal removal from an aqueous environment using an eco-friendly and sustainable adsorbent. *Sci. Rep.* **10**(1), 16453. 10.1038/s41598-020-73570-7 (2020). 33020581 10.1038/s41598-020-73570-7 PMC7536411

